# Every inch a finch: a commentary on Grant (1993) ‘Hybridization of Darwin's finches on Isla Daphne Major, Galapagos’

**DOI:** 10.1098/rstb.2014.0287

**Published:** 2015-04-19

**Authors:** Graham Bell

**Affiliations:** Biology Department, McGill University, 1205 avenue Docteur Penfield, Montreal, Quebec, Canada H3A 1B1

**Keywords:** hybridization, species, ecological speciation, sexual isolation, mating barrier, Darwin's finches

## Abstract

One of the most familiar features of the natural world is that most animals and plants fall into distinct categories known as species. The attempt to understand the nature of species and the origin of new species was the enterprise that drove the early development of evolutionary biology and has continued to be a major focus of research. Individuals belonging to the same species usually share a distinctive appearance and way of life, and they can mate together successfully and produce viable offspring. New species may evolve, therefore, either through ecological divergence or through sexual isolation. The balance between these processes will depend on the extent of hybridization, especially in the early stages of divergence. Detecting and measuring hybridization in natural populations, however, requires intensive, long-term field programmes that are seldom undertaken, leaving a gap in our understanding of species formation. The finch community of a small, isolated island in the Galapagos provided an opportunity to discover how frequently hybridization takes place between closely related species in a pristine location, and Peter Grant's paper, published in *Philosophical Transactions B* in 1993, reports the observations that he and his collaborators made during the first 20 years of what is now one of the classical studies of evolution in action. This commentary was written to celebrate the 350th anniversary of the journal *Philosophical Transactions of the Royal Society*.

## 1. Introduction

Daphne Major is a tiny island, not even 40 ha in area, of the Galapagos archipelago, which lies in the Pacific Ocean more than 1000 km off the coast of South America. Very few species have discovered a place so small and remote, and the only resident vertebrates apart from lizards are several hundred small birds in the genus *Geospiza*, which make their nests in the prickly pear cactus, *Opuntia*. These are the famous ‘Darwin's finches', which gave Darwin food for thought when he encountered them during the voyage of the Beagle. They are remarkable for their diversity of form, and in particular for variation in the shape of the beak, which is related in some degree to their diet. Two species are common on the island. One is the cactus finch, *G. scandens*, which has a rather long, slender beak and feeds mainly on the flowers and fruit of *Opuntia*. The other is the medium ground finch, *G. fortis*, which has a shorter, blunter beak and eats the small, soft seeds of shrubs such as *Chamaesyce*. There are also a few individuals of the small ground finch, *G. fuliginosa*, and of the large ground finch, *G. magnirostris*, which has a large, stout beak and is able to crack the hard seeds of plants such as *Tribulus*. Their diets overlap quite broadly, however, and vary over the year, and between years, in response to the availability of different seed crops. Their morphology is also variable; for example, the *fortis* of Daphne Major have beaks that are somewhat less robust than those of *fortis* individuals on other islands. Individuals that seem to be intermediate between species are also occasionally found. Nevertheless, the six species of *Geospiza* that have been recognized throughout the archipelago—together with eight other species of finch in closely related genera—vary consistently in morphology and ecology, even though their differences are far from absolute.

Darwin was impressed by the finches because all of them are endemic to the Galapagos. The existence of so many distinct forms, in a remote location where the familiar mainland birds were absent, seemed to contradict the universally accepted ideas of his time about the living world, and required a radically different interpretation. The idea that Darwin set out to overthrow, in part because of his observations on the Galapagos Islands, was the privileged status of the species. Before his time, a catalogue of species, building on the schemes of Linnaeus and Ray, was the main goal of natural history, because it would exhibit the plan of the world and thereby reveal the mind of God. This enterprise is credible only if species are fixed and immutable, which is what everyday experience seems to show: a cat is a cat, and has been since the time of the Pyramids. Darwin made the astonishing claim that, on the contrary, species are no more than strongly marked varieties, so that, just as one variety may admittedly give rise to another, so may any species arise by the transformation of an ancestor through a series of intermediate stages. At some point in this process, two lineages descending from a common ancestor become sufficiently distinct in morphology and ecology that they are given different names, but how this point is identified serves convenience rather than principle.

The extreme gradualism that is characteristic of Darwin's view of nature was widely accepted at the time with regard to the adaptation of species to new ways of life. Natural selection and sexual selection were thereby established as the principal agents of evolutionary modification, although they were viewed as acting only very weakly over very long periods of time (e.g. [1, p. 24]). (The emphasis on very slow change may have been necessary to gain the general acceptance of selection as the mechanism of evolution, but in many ways it impeded the development of evolutionary biology, especially by discouraging experimental studies of evolution, in the field or in the laboratory, for the best part of a century.) Darwin's views on speciation were less influential, and by the middle of the twentieth century they had been largely replaced by a new school of thought that emphasized sexual isolation rather than ecological distinctiveness as the criterion for the species boundary. The discrete nature of sexual isolation meant that it could be used, in principle at least, to define a unique set of individuals as constituting a species. In this way, the species re-emerged as the only natural category of classification: races and varieties were too loosely defined to have a consistent meaning, while the membership of genera, families and other Linnean categories was a matter of subjective judgement.

These two schools provided different interpretations of hybridization, and thereby of phylogeny. In the older, Darwinian tradition, hybrids were commonplace and expected: races and varieties often interbreed, and species will naturally do the same, although with lesser frequency as they become progressively more distinct. In the same way, hybrids are often completely viable and fertile, although they need not be, especially if the parents are dissimilar. The phylogenetic tree of a group of related species will show branching, caused by divergent natural section, but also anastomosis, caused by hybridization, at least until lineages become widely divergent. According to the newer view, hybrids are rare and regrettable instances in which the sexual barrier between species has been breached, perhaps as the consequence of some recent environmental change. Most hybrids are markedly inferior to either parent, and sexual isolation is thereby reinforced, through selection against inappropriate mating. The phylogenetic tree is strictly branching because hybrids are too rare or too feeble to make any appreciable contribution to it. This view is much clearer and more elegant that the rather vague notion of species inherent in the older tradition, and by the 1960s the newer had silently replaced the older.

**Figure 1.**
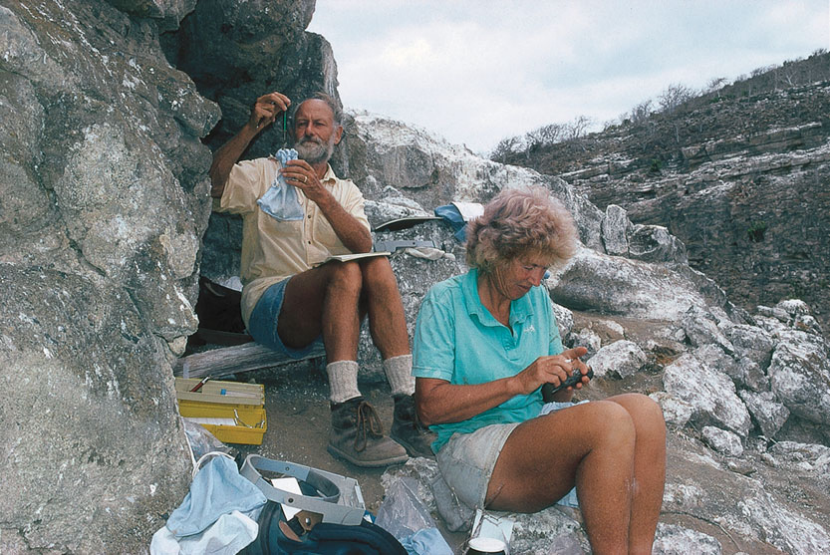
Peter and Rosemary Grant. Photograph kindly supplied by Peter Grant.

This is a simplification, of course, perhaps an outrageous simplification, of the labyrinthine and occasionally acrimonious debates about the ‘species concept’ that have continued down to the present day (without, in my opinion, adding much of substance to our understanding of evolution). I think it is fair, however, to contrast these two views of species, and to conclude that sexual isolation and strictly branching phylogenies have been broadly accepted as the leading features of species formation, at least since the publication of Ernst Mayr's magisterial tome (as I think it must be described) in 1963 [2]. Nevertheless, there were a few exceptional situations that seemed to support a more nuanced interpretation. One of these began to take shape when Peter and Rosemary Grant landed on Daphne Major in 1973 to begin a detailed study of its resident finches (figure 1).

## 2. The finch radiation on the Galapagos archipelago

**Figure 2.**
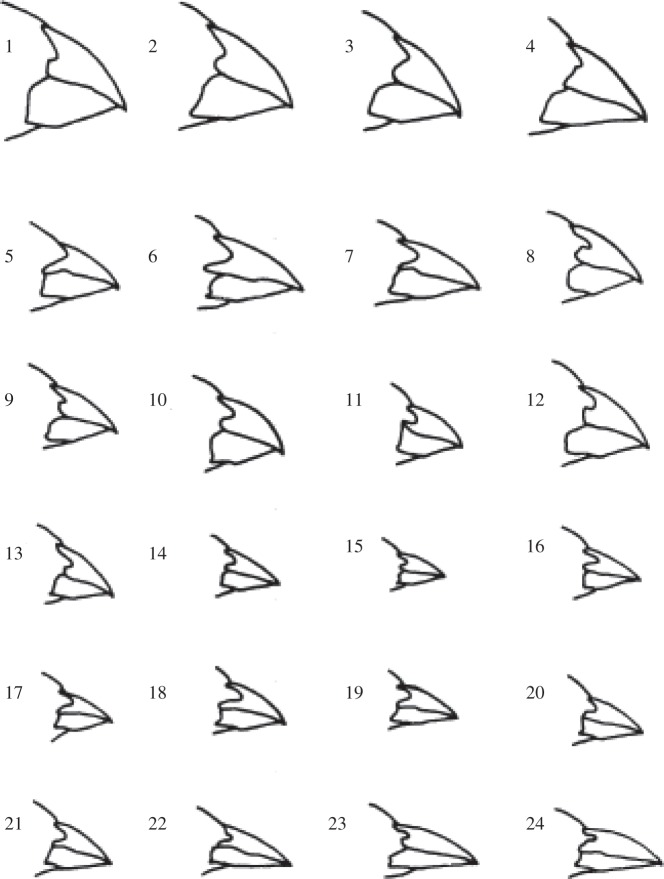
Gradation in beak size and shape of selected males of the six *Geospiza* species. Reprinted from Abbott *et al.* [3]. Republished with permission from the Ecological Society of America; permission conveyed through Copyright Clearance Center, Inc.

There are 13 named species of finch endemic to the islands of the Galapagos: five species of tree finch, *Camarhynchus*; the warbler finch, *Certhidea*; the vegetarian finch, *Platyspiza*; and the six species of ground finch, *Geospiza*. The group is often cited as a classical example of adaptive radiation, with a very wide range of morphological variation. In particular, there is extensive variation in beak shape, from large, broad, stout beaks in seed-eating species to slender, pointed beaks in insect-eating species (figure 2). The association of morphology with diet immediately suggests a mechanism for the adaptive radiation of this group from an unspecialized ancestor arriving in this remote archipelago.

**Figure 3.**
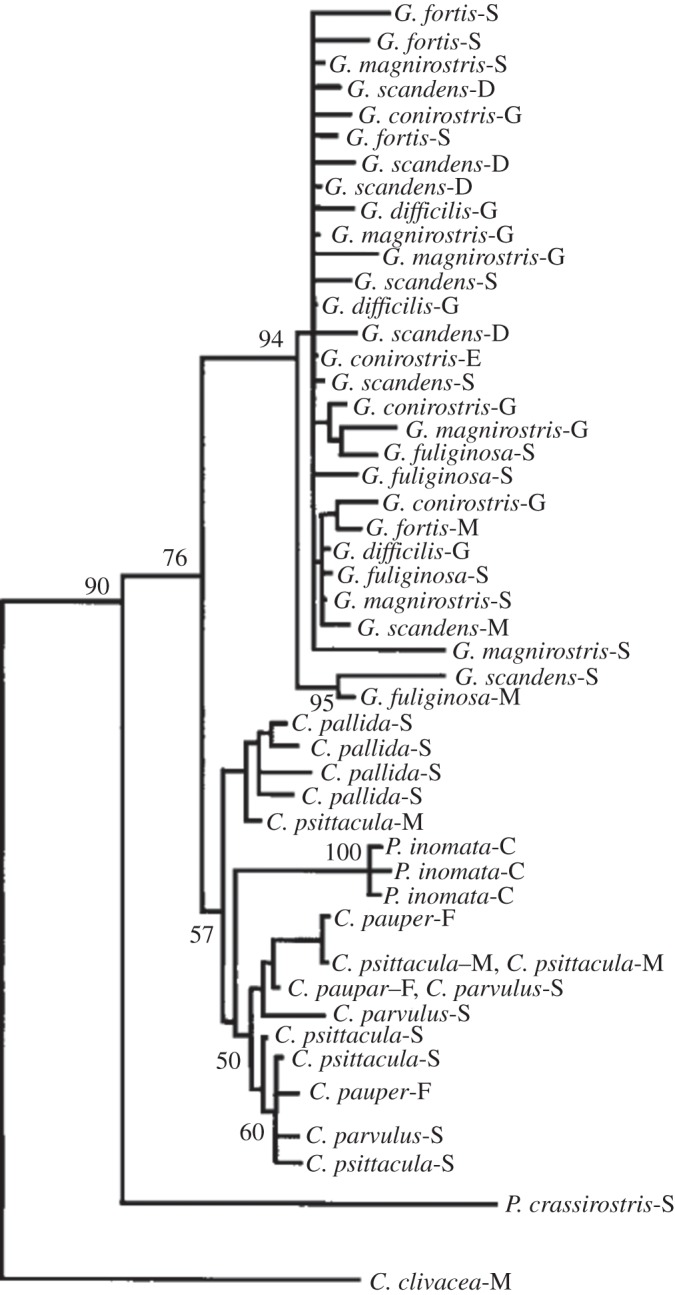
Neighbour-joining phylogenetic tree of *Geospiza* derived from mitochondrial DNA sequences (fig. 1 of [8], which was redrawn from fig. 1 of [7]).

The usual depiction of this radiation, however, as a phylogenetic tree branching into morphologically distinct species, is not wholly consistent with the rather limited DNA surveys that have been reported. Individual birds can indeed always be placed unequivocally in a genus, wherever they have been collected, and there is ample evidence that the genera are reciprocally monophyletic (i.e. all species within a genus have a common ancestor that is not the ancestor of any species in another genus). For species within a genus, and particularly for the species of *Geospiza*, the situation is different. Individuals which are assigned to different species often overlap in morphology and diet; individuals assigned to the same species may vary among islands; and it is often correspondingly difficult to assign an individual with confidence to any given species. Indeed, there may be greater genetic divergence between populations of a single species on different islands than between nominate species [4–7]. Consequently, the estimated phylogeny does not consistently recover the canonical species. The species assignment of an individual cannot be predicted from mitochondrial DNA data (e.g. [7]). Indeed, with respect to mitochondrial DNA sequences, a sample from all six *Geospiza* species is not very different from a sample from a single outcrossed population ([8]; figure 3). Hence, morphological variation in this group has not yet condensed into fully discrete, permanently isolated lineages. Instead, it is kept in flux by a combination of four processes: ecological divergence, hybridization, geographical variation and dispersal.

Ecological divergence evolves through selection for specialization on different food items. There is some evidence from detailed field studies that diet correlates with beak shape [9], although the correlation is not very strong or consistent, except that individuals with large stout beaks can handle large hard seeds and thereby have a broader diet [3]. The most convincing evidence that beak size and shape constrains diet is the shift in average phenotype that occurs when the vegetation changes in response to rainfall. The seed supply is reduced by severe drought, and the limited amount of small, soft seeds is soon exhausted; consequently, selection favours birds with stout beaks able to crack the large, hard seeds that remain. Conversely, the profusion of small, soft seeds in years of heavy rainfall reverses the direction of selection to favour birds with smaller and more slender beaks. The observation of fluctuating selection over drier and wetter years [10–13] shows how beak morphology is related to diet and can shift in response to changes in seed quality.

Ecological divergence will be obstructed by hybridization. As hybrids are morphologically intermediate between their parents they may be ecologically intermediate too, and in consequence may actually survive better than either parental type when the environment changes [11]. In this way, hybridization may actually facilitate the morphological response to shifts in seed supply [14], although it does not necessarily follow that hybridization is itself an adaptive response to environmental change.

There is also geographical variation in morphology among islands, as figure 2 illustrates. The *fortis* of Daphne Major, for example, have smaller, shallower beaks than *fortis* individuals on other islands. Some of this variation may be attributable to differences in vegetation among islands, as Grant & Grant [15] suggest for *G. conirostris*. Whatever be the cause, it implies that dispersal may affect the amount of variation expressed by a particular population. The populations on Daphne, for example, might be disproportionately affected by immigration from the much larger nearby island of Santa Cruz. The phenotypic distinctiveness of the Daphne *fortis*, however, suggests that they are largely isolated from neighbouring populations on other islands, with only a low level of immigration. Direct observations of ringed birds have confirmed that there is only a low level of immigration (≤1 individual per generation) of *fortis* and *scandens* to Daphne [16].

The consequence of the contending processes of selection, hybridization and dispersal is that phylogenetic studies of *Geospiza* have not produced a simple, strictly nested tree. Instead, individuals assigned to the same species are not all grouped together in the same lineage but rather appear at different places in the tree, indicating that the species is not monophyletic (figure 3). This implies that characters such as ‘stout beak capable of cracking hard seeds' have not evolved once only, uniquely marking a single lineage, but have rather evolved repeatedly, in different lineages and often on different islands. On each occasion, the outcome is a morphologically distinct type that might in the course of time evolve into a permanently separate lineage (as exemplified by the genera), but that is usually prevented from doing so by dispersal and hybridization. On a larger scale, this results in a swarm of ecotypes representing incipient species, held apart by divergent selection but still united by occasional hybridization.

## 3. Hybridization on Daphne Major

Efforts to interpret the radiation of Darwin's finches on the Galapagos Islands mirrored the larger debate about the nature of species. Earlier studies attributed a good deal of the extensive morphological variation in the group to hybridization between rather loosely defined species (e.g. [17]). Later studies emphasized the role of ecological competition in generating divergent natural selection for specialized diets, resulting in distinctive morphology (e.g. [18]). It is easy to appreciate with hindsight that the issue could not be satisfactorily resolved without a detailed, long-term survey that would not only detect hybridization but would also be capable of estimating its frequency and its effects. This is what the Grants, working with an exceptionally talented group of students and collaborators, set out to do.

Daphne Major is no more than 500 m long in any direction, and supports a few hundred finches in most years. The populations of the four species of *Geospiza* found on the island are morphologically distinctive with respect to beak shape, and can be reliably diagnosed by a combination of beak depth and beak length. There are no predators, and the birds are quite tame. Almost every individual can be captured and uniquely tagged with leg bands. The nests of almost all breeding pairs can be visited, and the number, survival and subsequent fate of their progeny can be recorded. After two decades of work, enough information had accumulated to provide a complete picture of the demography, diet and behaviour of the finches, including the vexed question of hybridization, which was the subject of the landmark 1993 article in the *Philosophical Transactions of the Royal Society* [19].

**Figure 4.**
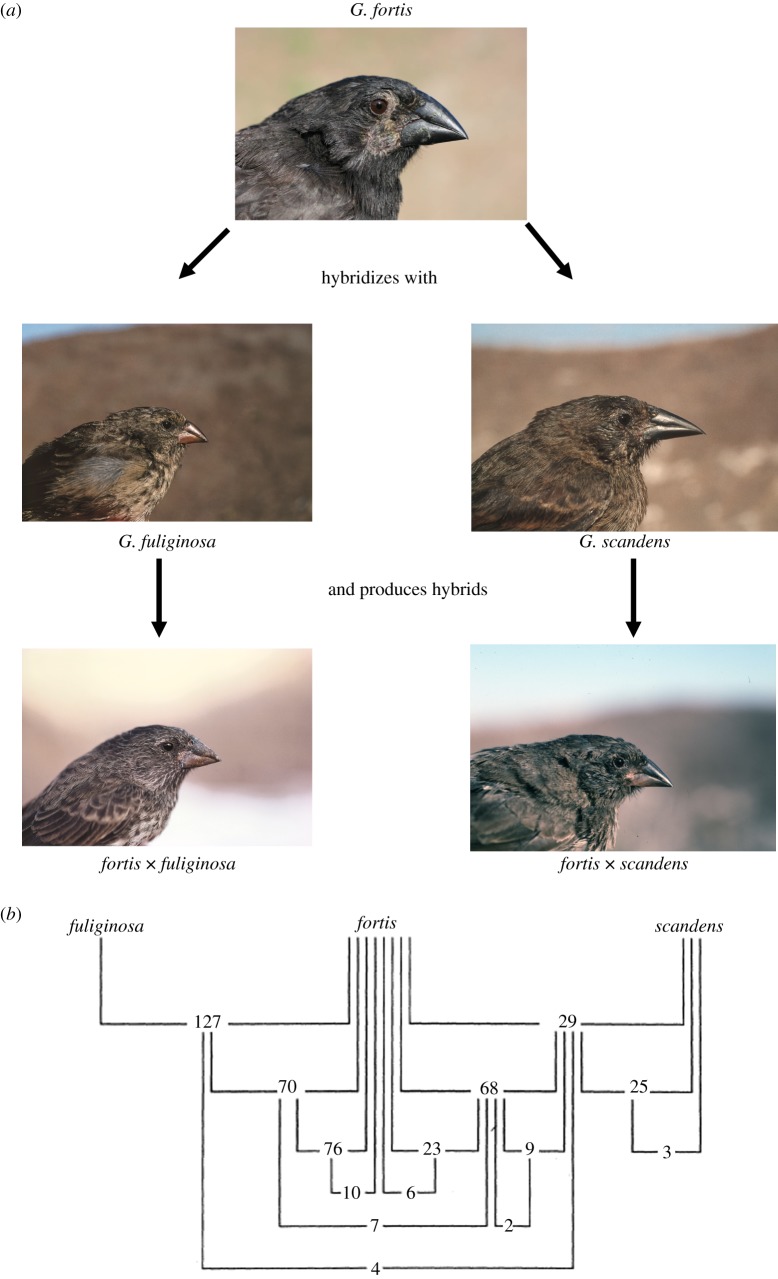
Hybridization of *Geospiza* species on Daphne Major. (*a*) Hybridizing species and the F1 hybrids (fig. 7 of Grant [19], recreated using images supplied by Peter Grant). (*b*) Number of fledglings produced by interspecific crosses and hybrid pairs (fig. 8 of [19]).

The first result of the survey was that hybridization occurred sufficiently often for its frequency to be reliably estimated (figure 4). Indeed, hybridization was common at the level of nominate species: *fortis* individuals mated with both *fuliginosa* and *scandens*, and their hybrid offspring mated among themselves and with the parental types. At the same time, hybridization was rare at the level of individuals: only a few percent of all offspring are hybrids. A similar result had previously been obtained for a different community of finches on Isla Genovesa, where about 1% of all offspring were hybrids between *G. conirostris* and either *G. magnirostris* or *G. difficilis* [20]. Hence, some low rate of hybridization seems to be widespread among species of *Geospiza*. This would explain why individuals can usually be assigned to one species or another on the basis of morphology, whereas related species are genetically more similar when on the same island than when on different islands [20].

The second striking result was that the success of matings between individuals of different species, gauged by the number of fledglings per clutch, was just as high as the success of matings between conspecific individuals. Moreover, matings between hybrids, or backcrosses of hybrids to the parental species, showed no consistent sign of reduced fitness. The admixture of *fortis* and *scandens* genomes, or *fortis* and *fuliginosa* genomes, in any proportion, had no detectable effect on the vigour of offspring. Hence, the ecological differences between species were not being maintained by selection against inviable intermediate types, at least after the El Niño event of 1983 that altered the vegetation of the island.

The pattern that the Grants and their collaborators discovered on Daphne Major, then, was a community of ecologically specialized groups that occasionally interbred to produce fully viable hybrids. Without divergent selection to maintain their distinctiveness, these groups should slowly coalesce, and this seems to be happening. Twenty years after the 1993 paper, the morphological differences between *fortis* and *scandens* have diminished appreciably, and a visit in mid-century might find only a single type, with no hint of the diversity that had once existed [22].

## 4. The rise of ‘ecological speciation’

There has been a long-running controversy in evolutionary biology about the possibility of ‘sympatric speciation’, which means the formation of two species from a single ancestral species in the same locality, where individuals can freely intermingle. It formed, so to speak, the left wing of speciation theory, in contrast to the more conventional right wing of allopatric speciation, which required geographical isolation between the diverging populations until they were completely sexually isolated. During the 1990s, sympatric speciation began to be supplemented, and to some degree supplanted, by the theory of ecological speciation, which is the view that ecological divergence is the primary stage in species formation, often but not necessarily occurring between sympatric populations. Ecological speciation was powerfully supported by new field studies, especially the extensive investigations of morphological divergence associated with diet in fish such as sticklebacks and whitefish in northern lakes. These frequently evolve into two sharply demarcated ecotypes, a smaller and more gracile type living in the open water and a stouter benthic type foraging in the littoral zone, that coexist in the same lake. This differentiation evolves rapidly and has been observed repeatedly in sticklebacks [23], whitefish [24] and other postglacial fish populations (e.g. smelt, *Osmerus* [25]). Although hybrids are produced and are viable, they are often inferior to their parents because of their intermediate ecological attributes (in sticklebacks [26]) or partial genetic incompatibility during development (in whitefish [27]).

The theory of ecological specialization is opposed to the view that sexual isolation comes first, so that multilocus genetic divergence can occur. Without sexual isolation, the randomizing effect of recombination effaces divergent specialization [28]; this is neatly captured by the simple model of Kirkpatrick & Ravigné [29]. This theory requires that hybridization is rare on an individual basis, but allows that it may be common at the level of ecotypes; that is, any two ecotypes may occasionally interbreed, provided that almost all individuals mate with their own ecotype. In this case, divergent specialization can be maintained, provided that, roughly speaking, the rate of selection acting against maladapted types is greater than the rate of hybridization.

The 1993 paper makes it clear that Darwin's finches are an instance of ecological speciation in the presence of hybridization. The species diverge morphologically, through natural selection for ecological specialization, in the same isolated archipelago, if not generally on the same island. Most individuals mate with another individual of the same species, but hybridization occurs occasionally. The hybrids may be fully viable despite—or sometimes because of—their intermediate morphology, or they may be much less fit than either parent, depending on the state of the environment. Divergent selection tends to maintain the morphological differences between nominate species, while hybridization tends to erode them. Depending on circumstances, species can be forced further apart or brought closer together, even to the point of losing their separate identities.

**Figure 5.**
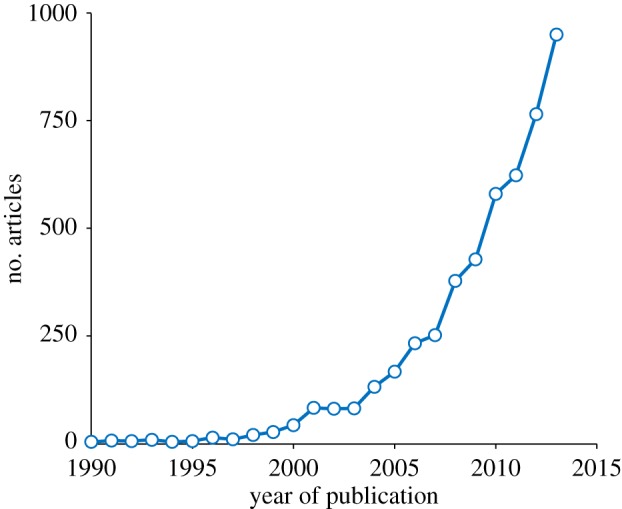
Trend in number of articles referring to ‘ecological speciation’ published in the last 20 years. Source: Google Scholar.

Building on these results, the research programme of the Grants has provided us with one of the classic accounts of evolution in action, revealing how natural selection routinely initiates the process of species formation. More than that, the 1993 paper marks the point at which interest in ecological speciation began to increase exponentially. At the time of its publication, only 5–10 papers per year referred to ecological speciation; by the end of the decade this number had increased to 30–40; at present about a thousand papers a year are published on this theme (figure 5). The painstaking, long-continued observations on a small, remote island have indeed borne abundant fruit.

The truth of the matter is likely to be, as usual, somewhere in between the extreme views of exclusively ecological and exclusively sexual divergence. We now understand that the phylogenies of sexual organisms are not the strictly branching trees of the classical school; but nor are they the free-for-all of reticulate evolution in bacteria exchanging genes by horizontal transfer. Hybridization is most frequent during the early stages of species formation, becomes less frequent as nascent species diverge and eventually ceases completely. The most general representation of a phylogeny, on this view, is a branching tree with sparse reticulation, largely confined to the diverging lineages close to the most recent common ancestor of sister taxa.

## 5. Saltation on Daphne

The contrast between strictly branching and basally reticulate phylogenies does not by any means exhaust the controversies about species formation; nor does it exhaust the lessons to be learned from the finches of Daphne Major. The suggestion that new species are most likely to arise suddenly in small populations, for example, those on the periphery of a species' range, has been called ‘peripatric speciation’ [30]. This might involve some radical change in genome structure that could only be fixed in small populations [31], although this idea has been strongly criticized [32]. The ecological idiosyncrasies and the constraints on mating that might occur in small populations, however, could provide a suitable arena for the initial stages of species formation—if only a convincing example could be found.

The most formidable obstacle faced by a sexual theory of speciation is the necessity for reciprocal change in sexual signal generation and signal reception between genders in order to produce a sexually compatible population isolated from its ancestor. If a mutant with an altered signal appears, it is unlikely to have a novel sexual identity; instead, it is almost certain to be incapable of mating. Even if by rare chance it encounters a reciprocally altered partner, their offspring will be rare types in the population as a whole, unlikely to meet and correspondingly unlikely to found a sexually distinct lineage. A sexually distinct lineage is likely to most emerge when mating occurs predominantly within small groups of close relatives, so that mating partners with novel sexual compatibility, created by a rare reciprocal shift of signal generation and reception, are kept together.

A reciprocal shift in sexual compatibility will readily arise, however, when mating preferences are governed by events during development that are shared by sibs. In many birds, including the ground finches, male nestlings learn the song of their father, and females learn to be attracted by this song. When the father's song differs from that sung by other males, brothers and sisters are predisposed to mate together, because they are likely to be rejected by, or are not attracted to, unrelated birds. If the population is restricted to a small area—such as Daphne Major—adult sibs may encounter one another often enough to form a sexually isolated lineage that is perpetuated by the heritability of male song, whether genetic or cultural.

**Figure 6.**
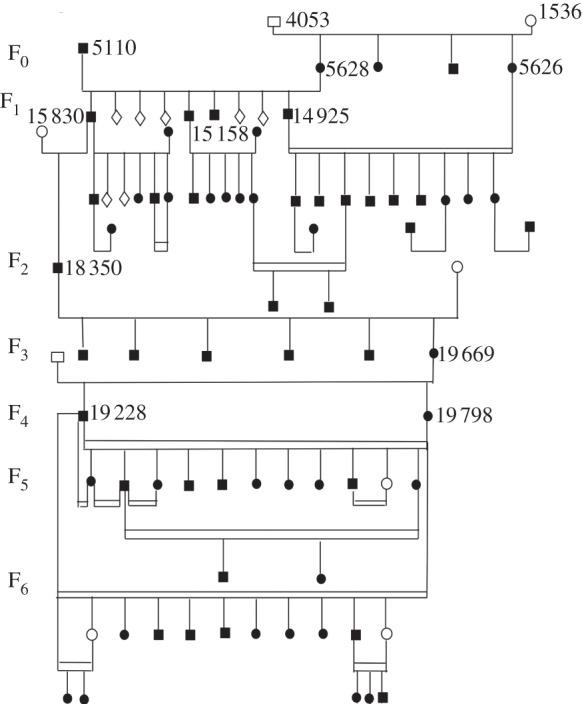
Pedigree of an immigrant *G. fortis* male (5110) with a line of descent to an exclusively inbreeding (endogamous) group (fig. 1 of [35]).

The possibility of rapid speciation in birds through sexual isolation mediated by song was clearly recognized by the Grants [33], and by good fortune they actually observed such an event, and were able to trace its consequences. A somewhat aberrant *fortis* male, almost certainly with some *scandens* ancestry, arrived on Daphne Major in 1981, probably from the nearby, much larger island of Santa Cruz. It was an unusually stout individual with a broad, pointed beak, and it sang an unusual song. For the next 28 years, its descendants were traced generation by generation. In the fourth generation, the finch population was depleted by a severe drought, and the immigrant lineage was reduced to a single pair of brother and sister. From this point on, young birds heard only the song peculiar to their lineage, and in consequence mated when adult only among themselves, and not with individuals from the surrounding *fortis* population. They occupied clusters of territories, within auditory range of one another. They also retained the unusual beak morphology of their ancestor, suggesting some degree of ecological specialization. In short, ecological speciation and sexual speciation have in this case acted in concert; and in consequence this lineage had proceeded some distance down the path that eventually leads to distinctive ecological specialization and complete sexual isolation [22,34] (figure 6).

In all likelihood, it will proceed no further. Such a very small population is inevitably vulnerable to accidents and environmental fluctuations, and is likely to become extinct in the near future. It seems most unlikely, however, that it represents a very rare event. For such an event to be observed in one of the very few wild populations that have ever been scrutinized so thoroughly that it could be observed suggests rather strongly that similar events are occurring rather frequently in other populations where they would never be detected. This argument certainly falls short of proof, but it does raise the possibility that nascent species, far from arising only in exceptional circumstances at long intervals of time, may instead continually arise at a measurable frequency, at least in some kinds of organism, even though most become extinct before expanding enough to be noticed. ‘Speciation appears to be easy; the intermediate stages are all around us' [36, p. 2980].

I shall conclude with an historical footnote. To understand the phenotypic variation among the finches of Daphne Major, it was important to identify possible sources of immigrants. Santa Cruz was the obvious possibility; but there were others, including the very small nearby island of Daphne Minor. Unfortunately, this sheer-sided plug of lava rises abruptly from the ocean without offering any feasible landfall. The Grants therefore recruited a McGill colleague, Howard Bussey, as the leader of a small party to establish a fixed rope as a route to the interior of the island. The overhangs of soft, crumbling rock (‘hardish as cheeses go’) made this a hazardous enterprise [37], but after a series of adventures (including an unplanned descent, fortunately on a top rope) the interior was reached and the resident finches surveyed. As it turned out, only a few *scandens* had migrated there from Daphne Major, and no *fortis*; in the reverse direction, two *fortis* from Daphne Minor were seen on Daphne Major but did not breed [10,11]. The Grants and the climbing team were probably the first featherless bipeds to visit this inaccessible site; it was important to be sure.

## Author profile

**Graham Bell** is James McGill Professor in the Department of Biology in McGill University. He is an evolutionary biologist and ecologist who has worked on a broad range of questions. One of the most striking results of his research has been the discovery that selection in laboratory microcosms over relatively short periods of time can produce radical changes to the structure or way of life of organisms. These studies show that experimental evolution offers an effective technique for studying radical changes in the ecological attributes of organisms. In the last five years, his laboratory has also pioneered the experimental study of evolutionary rescue, the evolutionary adaptation of a population through natural selection to stressful conditions that would otherwise lead to extinction.

In 1977, Peter Grant took a sabbatical year from McGill University to continue his systematic observations of the very birds that first aroused Darwin's interest in the origin of species. At that time, universities provided temporary replacements for professors on sabbatical leave, which is why Graham Bell arrived at McGill in 1976, just before Peter Grant set off for the Galapagos, to take over his teaching duties during his absence. Sixteen years later, Bell was still at McGill and Peter Grant published the results of his investigation of hybridization in Darwin's finches.
